# Making a Virtue Out of Necessity: COVID-19 as a Catalyst for Applying Internet-Based Psychological Interventions for Informal Caregivers

**DOI:** 10.3389/fpsyg.2022.856016

**Published:** 2022-04-07

**Authors:** Michelle Semonella, Gerhard Andersson, Rachel Dekel, Giada Pietrabissa, Noa Vilchinsky

**Affiliations:** ^1^Department of Psychology, Bar-Ilan University, Ramat Gan, Israel; ^2^Department of Behavioral Sciences and Learning, Linköping University, Linköping, Sweden; ^3^Department of Biomedical and Clinical Sciences, Linköping University, Linköping, Sweden; ^4^Division of Psychiatry, Department of Clinical Neuroscience, Karolinska Institute, Stockholm, Sweden; ^5^School of Social Work, Bar-Ilan University, Ramat Gan, Israel; ^6^Department of Psychology, Catholic University of Milan, Milan, Italy; ^7^Psychology Research Laboratory, Istituto Auxologico Italiano - Istituto di Ricovero e Cura a Carattere Scientifico, Milan, Italy

**Keywords:** internet-based intervention, informal caregiver, clinical psychology, caregiver burden, self-help, COVID-19, digital mental health

Coronavirus disease 2019 (COVID-19), which dates from December 2019, has been recognized by the World Health Organization (WHO) as a global pandemic on March 11, 2020. In order to face the ongoing pandemic and prevent the spread of the virus, national governments worldwide imposed various restrictions (Sandford, 2020). Yet, lockdown, mobility restrictions, and fear of uncertainness put the mental wellbeing of many individuals at risk (Serafini et al., 2020). Many studies have reported on negative health outcomes during the COVID-19 outbreak, such as maladaptive and unhealthy behaviors (e.g., sedentary lifestyle, consumption of alcohol, or unhealthy diet) (Brooks et al., 2020), higher levels of anxiety, depressive symptoms, irritability (Pearman et al., 2020; Rossi et al., 2020), posttraumatic stress and psychological stress (Cooke et al., 2020), disinterest, poor health perception (Le and Nguyen, 2021), and concern for one's self and worry for the others (Khan et al., 2020), along with feeling lack of personal freedom and to be under constant emergency during the quarantine (Brooks et al., 2020; Durosini et al., 2021).

Particularly, some populations, due to their unique characteristics or societal role, have endured a worse impact than others. One such population which has gained relatively little attention in the literature, is informal caregivers (Budnick et al., 2021; Sheth et al., 2021). Informal caregivers are family members, neighbors, friends, or other non-kin who provide unpaid care to an older, frail, or ill person (Triantafillou et al., 2010). Above and beyond the fear of infection and economic difficulty, informal caregivers have experienced an extended burden due to their unique social role (Lorenz-Dant, 2020). Different studies conducted during the pandemic have identified three main challenges for those who provide informal care: 1. decreased routinely provided healthcare options, 2. emotional distress, and 3. economic stress (Glaser et al., 1967; Phillips et al., 2020; Sheth et al., 2021). The restrictive and preventive measures that have been put in place to stop the spread of the virus have given way to an increase in informal care duties due to the drastic reduction in the availability of family members and/or respite care (Schmidt et al., 2020), and diminished availability of volunteers and social services (Arnold et al., 2021).

Especially among non-cohabiting informal caregivers, there has been an increase in “competing” care needs. For example, the closing of schools has required informal caregivers who are also parents to manage the needs of both their children and their care recipients, making the role of informal caregivers even more stressful than in ordinary times (Rodrigues et al., 2021). The virus's high infectiousness has led to the fear of being infected and, consequently, to the infection of care receivers, and/or to caregivers being quarantined and unable to visit the person they are caring for (Cipolletta et al., 2021). Economic stressors, linked to job loss or a decrease in working hours, and consequently salaries, are also factors in this increased burden (Arnold et al., 2021). Clearly, the caregiving situation, which was challenging to begin with, has become even more complex. In addition, the restrictions posed on society have made it harder to be helped by means of traditional psychotherapy sessions which are usually delivered face-to face. Not only have caregivers experienced more distress and burnout, but the ways of helping them have also been made more challenging.

Indeed, many of these challenges cannot be overcome; nevertheless, we can help caregivers cope better *emotionally* with their situation. Any possible solution should take into account not only the challenges that informal caregivers have been facing due to the pandemic restrictions, but also the limitations which characterize their role in general, such as time limitations. Thus, caregivers will gain from interventions that help them cope with their challenges but also pay heed to the new imposed constraints. One such possible solution are digital-based interventions.

The pandemic has led to an increased interest in diverse digital-based solutions, comprising videocall meetings, telephone calls, and mobile applications that were shown to be effective even before the pandemic (Bertuzzi et al., 2021). In the specific context of relieving caregiver burden, the use of digital solutions has increased, along with caregivers' acceptance of and willingness to use them, and have been adopted by different healthcare entities to deliver services (e.g., videoconferencing, and mobile applications) (Blumenstyk, 2020; Bertuzzi et al., 2021).

Although there is great potential in the use of digital solutions for supporting caregivers (Hassan, 2020), these solutions are not necessarily optimally tailored to the specific needs (e.g., time constraints, anonymity) of informal caregivers (Semonella et al., 2020). For example, the need to schedule meetings in advance, and to be present synchronously in sessions, might be highly problematic for busy caregivers. Also, psychological support interventions which are delivered *via* telephone or videocall with an actual therapist might not provide the level of anonymity that caregivers prefer, due to issues of guilt or shame (Springate and Tremont, 2014; Woodford et al., 2018; Moudatsou et al., 2021). Thus, there is a need for interventions that can be delivered *via* ordinary technological devices (i.e., mobile phone or personal computer) that informal caregivers can use easily. In addition, such interventions should be monitored by a therapist in an asynchronous and anonymous manner. Hence, we need solutions that are feasible, suitable, and sustainable over time, above and beyond the demands of the current pandemic.

As such, we suggest using specific digital solutions, which have been termed internet-based interventions. Internet-based interventions consist of psychological support *via* a comprehensive internet-based platform including therapeutic modules, usually based on cognitive and behavioral therapy, and weekly therapeutic tasks that need to be completed *via* use of the computer (Andersson and Titov, 2014). Using internet-based interventions might address the three main challenges confronted by informal caregivers, in ordinary times as well as during the pandemic. First and foremost, different studies have shown the beneficial effects of internet-based interventions on informal caregivers' mental health, including reduction of depression, stress, anxiety, and burden (Egan et al., 2018; Sherifali et al., 2018). In addition internet-based interventions have been found to increase caregivers' self-efficacy and self-esteem (Ploeg et al., 2018) as well as their sense of competence, coping skills, and quality of life (Guay et al., 2017). In fact, internet-based interventions have been found to be as effective as other treatment formats (Andersson et al., 2013).

Moreover, although other digital solutions have been found to be beneficial for reducing caregivers' distress, particularly during COVID-19 (Bertuzzi et al., 2021), what makes internet-based interventions feasible is the fact that one has maximum time flexibility when using the platform. Such flexibility is most likely an advantage for caregivers, as finding additional time slots for face-to-face therapy might be impossible for them (Caregiving NAf, 2015). Also, unlike other digital solutions (i.e., calls *via* telephone or video conferencing platforms such as Skype, Zoom, Google Meet, etc.), which require synchronized participation of both patient and therapist, internet-based interventions allow clients to access the therapeutic platform whenever and wherever it suits them; they only need a computer/tablet/mobile phone and internet access (Johansson et al., 2021). It can be difficult and time-consuming for informal caregivers to reach clinics or therapists, especially for those who live far away from their therapist and/or clinic (Bei et al., 2020). By using internet-based interventions it is possible to reach therapists from a distance. Moreover, this specific way of delivering interventions offers a secure platform, allowing the maintenance of one's anonymity (Andersson, 2016). Pre-set reminders are being sent to informal caregivers to complete the modules and to check homework. Moreover, informal caregivers can communicate with the therapist using the platform when needed, with maximum time flexibility and anonymity. Finally, financial strain notwithstanding, it has been shown that internet-based interventions are more cost-effective than face-to-face treatment (Andersson and Titov, 2014; Kolovos et al., 2018).

Despite the benefits of using internet-based interventions within the informal caregiving context, healthcare professionals' acceptance hesitancy and other expected barriers did not, prior to the pandemic outbreak, allow for a full integration of internet-based interventions in clinical practice (Topooco et al., 2017). Further studies on informal caregivers should exploit the advantages of what has emerged from the current situation to shift the perspective in thinking and deliver therapeutic solutions to support informal caregivers around the world. For example, as part of a joint European consortium, an internet-delivered cognitive behavioral therapy (iCBT) intervention for informal caregivers has already been tested in Lithuania. The results of this study suggest that this internet-based intervention can be effective in reducing caregivers' burden, anxiety, depression, and stress, and in improving quality of life (Biliunaite et al., 2021). In light of this successful endeavor, we are currently evaluating the efficacy of a similar internet-based intervention for informal caregivers in Italy, a huge country with a substantial percentage of caregivers (ISTAT, 2018) and in which, surprisingly, no such solution has been provided before (Semonella et al., 2020).

In conclusion, beyond all the well-known negative outcomes of this pandemic, COVID-19 also appears to have been a catalyst for many positive advances, including the reduced antagonism toward digital tools, such as internet-based interventions (Wind et al., 2020). The current momentum should be harnessed for the development and implementation of internet-based interventions around the globe, especially for populations in great need of these state-of-the-art solutions, such as informal caregivers.

## Author Contributions

MS and NV wrote the first draft of the manuscript. GA, RD, and GP revised the manuscript and giving precious feedback and suggestions. All the authors contributed to the conceptualization of the manuscript. All authors contributed to the article and approved the submitted version.

## Funding

This opinion paper was undertaken as part of a Ph.D., thesis. The Ph.D., was funded by EC-funded Marie Sklodowska-Curie Innovative Training Network (H2020-MSCA-ITN-2018), Grant Agreement No. 814072. The funder has not had any role in the preparation of the manuscript.

## Conflict of Interest

The authors declare that the research was conducted in the absence of any commercial or financial relationships that could be construed as a potential conflict of interest.

## Publisher's Note

All claims expressed in this article are solely those of the authors and do not necessarily represent those of their affiliated organizations, or those of the publisher, the editors and the reviewers. Any product that may be evaluated in this article, or claim that may be made by its manufacturer, is not guaranteed or endorsed by the publisher.
